# A combination of traditional learning and e-learning can be more effective on radiological interpretation skills in medical students: a pre- and post-intervention study

**DOI:** 10.1186/s12909-016-0569-5

**Published:** 2016-02-03

**Authors:** Ali Salajegheh, Alborz Jahangiri, Elliot Dolan-Evans, Sahar Pakneshan

**Affiliations:** School of Medicine, Menzies Health Institute Queensland, Griffith University, Gold Coast Campus, Gold Coast, QLD 4222 Australia

**Keywords:** Blended learning, Radiology, Interpretation, Medical education

## Abstract

**Background:**

The ability to interpret an X-Ray is a vital skill for graduating medical students which guides clinicians towards accurate diagnosis and treatment of the patient. However, research has suggested that radiological interpretation skills are less than satisfactory in not only medical students, but also in residents and consultants.

**Methods:**

This study investigated the effectiveness of e-learning for the development of X-ray interpretation skills in pre-clinical medical students. Competencies in clinical X-Ray interpretation were assessed by comparison of pre- and post-intervention scores and one year follow up assessment, where the e-learning course was the ‘intervention’.

**Results:**

Our results demonstrate improved knowledge and skills in X-ray interpretation in students. Assessment of the post training students showed significantly higher scores than the scores of control group of students undertaking the same assessment at the same time.

**Conclusions:**

The development of the Internet and advances in multimedia technologies has paved the way for computer-assisted education. As more rural clinical schools are established the electronic delivery of radiology teaching through websites will become a necessity. The use of e-learning to deliver radiology tuition to medical students represents an exciting alternative and is an effective method of developing competency in radiological interpretation for medical students.

## Background

The ability to interpret an X-Ray is a vital skill for medical students, as this imaging modality guides the medical professional towards accurate diagnosis and treatment of a variety of conditions. However, radiological interpretation skills may be less than satisfactory in not only medical students, but also in registrars and consultants [1–9]. With the advancement of imaging technology, medicine is increasingly relying on the unsurpassed anatomical and pathological information provided by radiology, and medical students must be able to extract pertinent clinical information from radiological images.

Formal teaching time in medical school devoted to the interpretation of radiological images is lacking. The duration of specific, pre-clinical radiology teaching in Australian medical schools averages 4 h a year [10]. These statistics are disappointing, as imaging can be utilised as a dynamic teaching utility, demonstrating anatomy, pathology and physiology [11]. Radiology is typically introduced to medical students in their clinical years [12], when they are confronted with radiological images alongside technical questions from senior doctors. This format may be inadequate to ensure optimal radiology learning, and it may be more beneficial for both students and clinicians that medical students are introduced to radiology as a subject earlier in their training. However, due to the 4-year postgraduate medical degree prevailing in Australian medical schools, it may be unfeasible to introduce additional didactic teaching for radiology to an already condensed program.

The use of e-learning to deliver radiology tuition represents an exciting alternative. E-learning allows students to perfect their skills in a stress-free, non-judgemental environment [13]. E-learning increases the possibilities for more dynamic interactivity and feedback between teacher and student [14], whilst making education generally more accessible and allowing the learner to work through the content at their own pace [15]. Radiology lends itself particularly well to implementation on a computer-based format due to the highly visual nature of the content [16].

One of the current issues with radiology-focused educational materials that are available on-line is that they are generally not standardised nor specifically designed to test determined competencies. Medical students have recognised these flaws in current, freely available online radiology modules [17]. However, when e-learning has been specifically designed for medical students and constructed in an interactive manner, this learning modality has been effective in developing competency in radiological interpretation for medical students [18]. Due to the great promise for e-learning teaching modalities to revolutionise education in the future [19], the primary aim of this study was to further investigate the effectiveness of e-learning for the development of x-ray interpretation skills in pre-clinical medical students.

## Methods

### Subjects

Students were first and second year students from the Griffith University Bachelor of Medicine/Bachelor of Surgery Program, a 4-year graduate entry program with admission via GAMSAT, grade-point average and interview hurdles. First year students who participated in the study (57/152; 37 % response) were allocated to the ‘intervention group’ receiving the e-learning course; whilst current second year students who participated (66/148; 44 % response) were the ‘control group’ who did not receive e-learning. Both intervention group and control group participated in our traditional formal radiological education activity that included an introductory lecture about how to interpret a chest X-ray followed by a small-group practical workshop where they practiced and developed their skills by studying different chest X-rays. Participants all gave informed consent prior to the experiment via an online form, which was approved by the Griffith University ethics committee for its conduct and publication (MED/23/12/HREC).

### Intervention

The e-learning package (intervention) was developed to enhance students’ competencies in clinical X-ray interpretation. The package was developed as an online interactive webpage within the university website. The program was freely accessible online via the Blackboard suite with the use of the student’s university username and password. On front page of the online package ,objectives of the activity were described. This included common understanding of the techniques used for obtaining a proper chest X-ray. Terms such as inspiration, penetration, and rotation were explained followed by examples of different images of those terms. Anatomy of the respiratory system was then described and demonstrated with figures of overlapping images of different elements of respiration with the actual chest X-ray. A common method of interpretation was then introduced to the students to develop a consistent and thorough technique for reading images and learn how the silhouette sign can help localize pathology. The final stage of the online package was demonstration of interactive images of different lung and respiratory system pathological lesions that could be recognised and identified by X-ray. Each image had a phase of plan image where no information was provided and then an image with full description and arrows showing the location, shape and forms of the pathology. From a long list of diseases presented pulmonary oedema, tuberculosis, fractured rib, diaphragmatic hernia and solitary pulmonary nodules were few of the popular examples. The X-ray e-learning package allowed pupils to explore the relevant anatomy and pathology that can be demonstrated with radiological imaging in a series of high-quality X-Ray images and associated teaching material.

### Assessment

X-ray interpretation skills were assessed at three time points in the study through an online multiple-choice questionnaire of thirty questions, with four possible answers and 2 min to complete each question. Questions were designed by an expert in radiology and checked with multiple qualified confederates.

At the conclusion of the study, participants were invited to complete a questionnaire assessing their satisfaction with the e-learning course using a 5-point Likert scale, in relation to the quality, accessibility, effectiveness and organisation of the online package, especially in comparison with didactic lecture-based learning. Students were also asked to complete free text responses to aspects of the e-learning course.

### Structure

For the intervention group, the e-learning course was made available a week before the start of formal radiological teaching that included a lecture and one small-group workshop. Prior to being able to access the e-learning course, students were required to complete a pre-intervention questionnaire as detailed above. A week after the lecture and workshop classes, the intervention group was again administered a questionnaire (post-intervention assessment 1).

Participants in the control group completed the same questionnaire as the pre-intervention questionnaire of the intervention group. These participants had free access to the e-learning program after the completion of the study.

One year after the initial study, a follow up set of questionnaire was again administered to ensure that any meaningful variation in the marks collected from the control group were not affected by the time lapse of the delivered material in that cohort. Therefore, students in the intervention group were again invited to participate in another online multiple-choice questionnaire (post-intervention assessment 2).

### Data analysis

All data was entered in to the statistical analysis software, Statistical Package for Social Sciences (SPSS version 22.0, IBM, New York, NY, USA). Final data was analysed using paired and independent t-tests and ANOVA (using LSD correction). Significance threshold was taken at *p* ≤ 0.05.

## Results

### Response rate

For the first year cohort, fifty-seven students out of one hundred and fifty-seven participated in the pre-intervention test, a response rate of 37.5 %. Forty-two students then participated in the post-intervention test 1, a response rate of 27.6 % and Forty-one students participated in the post-intervention test 2, a response rate of 26.9 %.

For the second year cohort, sixty-six students out of one hundred and forty-two participated in the test, a response rate of 44 %.

### Assessment results

Scores from the MCQ tests for the control and intervention groups are presented in Table 1. The results indicate that students in the intervention group improved their X-ray interpretation skills from prior to the e-learning course (mean score of 57.9 %) to after implementation (mean score of 70 %), and this improvement was found to be statistically significant (*p* < 0.05).

**Table 1:** X-Ray interpretation scores (presented as percentage with ± standard deviation)

	Pre-intervention MCQ	Post-intervention MCQ	One year after intervention
Intervention group	57.9 ± 9.7	70 ± 4.6	66.7 ± 5.8
Control group	60.3 ± 9.6	N/A	N/A

Comparison of the scores (presented as percentage with ± standard deviation), of the participants in control group (*n* = 66) with the study cohort with intervention (pre-intervention, *n* = 57, post-intervention, *n* = 42 and a year after intervention, *n* = 41)

Following e-learning, students in the intervention group scored higher in the MCQ test compared to participants in the control group who did not receive the e-learning course. Post-intervention scores for the intervention group (mean score of 70 %) was higher than the solitary MCQ for the control group (mean score of 63.2 %), and this difference was statistically significant (*p* < 0.05).

**Fig. 1:**
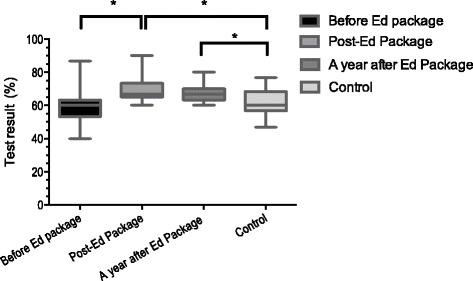
On-line test results in the study group before and after intervention compared with control cohort. Test result in the study group before and after delivery of the educational package (Ed Package) and one year after the activity in comparison with similar control cohort without the online educational package showed a significant improvement in the test scores after the intervention in the study group (*p* < 0.05). The one year after intervention test revealed that the intervention helped students maintain their knowledge significantly better compared to the control group without intervention (*p* < 0.05). Asterisks (*) show the significant changes

Furthermore, the follow up MCQ test after one year revealed that although there was a slight insignificant drop in the mean score of the second post-intervention assessment (mean score of 66.7 %), students in the intervention group maintained their X-ray interpretation skills and still achieved significantly higher marks that the control group (*p* < 0.05) (Fig. 1).

### Feedback results

Tables 2 and 3 show the responses to the Likert scale statements from the first-year students (intervention group) and second year students (control group). Analysis of participants’ answers demonstrates that a majority of students (65.9 %) found the on-line course to be effective in guiding them through the learning process. An overwhelming proportion (86.4 %) of the students also found the X-ray online package to be well organised, and importantly, most of the students (71.4 %) indicated that the e-learning modality engaged them in learning.

**Table 2:** Responses to 14 Likert scale questions related to interpretation of chest X-ray educational activity

Likert scale questions	Mean		Significance
	Study group	Control	
1. How effective was the large group resource session in preparing you for what you had to do in this session?	3.50	1.91	NS
2. How effective was the lecturer in relating the large group resource session’s learning objectives to the clinical practice workshop?	3.55	3.96	0.003
3. How effective was the lecturer in guiding you through the experiential learning process?	3.51	4.29	NS
4. My facilitator covered all the objectives of this session?	4.37	4.29	NS
5. My facilitator used approaches that helped me to learn?	4.56	4.47	NS
6. My facilitator was motivating and inspiring me to learn?	4.40	4.36	NS
7. My facilitator has highlighted the relevance of what I have to learn?	4.26	4.53	NS
8. My facilitator assessed my prior knowledge before explaining new material	3.72	4.28	NS
9. My facilitator was ensuring that I received feedback which helped me to learn?	4.05	4.30	NS
10. My facilitator explained the requirements and standards of work for excellence?	3.61	4.21	NS
11. My facilitator helped me to extend my knowledge understanding and skills (i.e. challenging me)	4.19	4.54	NS
12. My facilitator was helping me to learn in an organized, coherent and well-ordered manner?	4.24	4.51	NS
13. My facilitator was using feedback to improve his/her facilitation?	3.46	4.40	NS
14. My facilitator was effective in helping me to learn overall?	4.36	4.55	NS

**Table 3:** First year evaluation (intervention group) of the e-learning package

	Number of responders	Percentage of responders
The X-ray online package was well-organized (*n* = 44)		
Strongly agree	12	27.3 %
Agree	26	59.1 %
Neutral	4	9.1 %
Disagree	2	4.5 %
Strongly disagree	0	0 %
How effective was the X-ray on-line package in guiding you through the experiential learning process? (*n* = 44)		
Very effective	3	6.8 %
Effective	26	59.1 %
Neutral	7	15.9 %
Ineffective	8	18.2 %
Very ineffective	0	0 %
This component (X-ray interpretation on-line learning package) engaged me in learning (*n* = 44)		
Strongly agree	9	20.5 %
Agree	25	56.8 %
Neutral	8	18.2 %
Disagree	2	4.5 %
Strongly disagree	0	0 %
Overall, how effective was this component in helping you learn how to interpret X-rays? (*n* = 42)		
Very effective	11	26.2 %
Effective	19	45.2 %
Neutral	11	26.2 %
Ineffective	1	2.4 %
Very ineffective	0	0 %
Overall, I am satisfied with the quality of this learning activity (*n* = 42)		
Strongly agree	9	21.4 %
Agree	21	50 %
Neutral	9	21.4 %
Disagree	3	7.1 %
Strongly disagree	0	0 %

Arguably of most relevance to this study, results of the student feedback show that a large majority of students (71.4 %) felt that the e-learning package was effective in helping them learn the art of interpreting X-rays. 71.4 % of students were also satisfied with the quality of the online learning activity.

### Analysis of free text responses

Students were invited to comment on the most useful and engaging parts of the online learning package. In total, an average of thirty-two (32) students responded to the free text portion of the survey. There were three major themes that were raised by the students, where 31 % appreciated the resource, 15 % identified clinically relevant learning as the best aspect of the resource, and 12 % enjoyed the quantity of films available. Table 4 shows the key themes identified in the comments.

**Table 4:** Key themes identified from free text student responses for online-learning x-ray package

Key issue	Number of similar responses	Sample comments
Appreciated the resource	10	‘Thank you, I found this activity to be very useful’
Clinically relevant learning	5	‘I liked that the examples were given in the context of a patient’
Quantity of X-Rays	4	‘Lots of examples of Pathology on X-Ray
		I think that it’s important for 1st year med students as we just need to see more and more X-Rays’

## Discussion

Basic radiology interpretation is a key skill for medical students [20]. However, in most medical schools radiology training is not formally introduced until clinical rotations [11]. Prior studies have demonstrated less than satisfactory interpretation skills in medical students, which may contribute to a high rate of incorrect statements found in clinical radiology report [17]. Indeed, incompetence in x-ray interpretation has been shown to lead to management errors and adverse patient outcomes [16], which indicate that more effective training is needed in this vital area [4].

Acquisition of radiological skills can come from a number of sources [12]. Due to its highly visual nature, radiology lends itself particularly well to online learning on multimedia devices. Advances in multimedia technologies have eased time pressures on educators and has paved the way for computer-assisted education [16]. Evidence is also emerging that highlights the preference students have for computer-assisted learning when compared to reference books [21].

This study has demonstrated that learning radiology online is not only a more effective method, but also highly appreciated by students. Novice students improved significantly on x-ray interpretation scores following implementation of an e-learning x-ray interpretation course, scoring more highly on x-ray interpretation tests than students who did not receive e-learning. The students in the control group only experienced workshop and lecture teaching on x-ray interpretation, which the intervention group also received; and even though they were ahead in their studies by a year compared to the intervention group at the time of assessment, they performed worse than the group that received the online learning. This result is consistent with other evidence that has demonstrated implementation of online learning decreased the number of failing students [7], and that for radiography learning, students who experience computer-based learning score better in radiological assessment compared to ones who learn from a textbook [22, 23].

Three key themes emerged from the students’ free-text responses to the post-intervention survey: (1) students appreciated the online resource, (2) they found it clinically relevant, and (3) there was a sufficient quantity of X-rays to learn from. Other studies have also shown that students have a positive attitude towards on-line learning for radiology [24]. This feedback from students is very important as it indicates the level to which students will access, and continue to access, this learning medium to enhance their skills in radiological interpretation. As most medical students have access to a personal computer, students are comfortable using computer-based information resources for their learning. A majority of students may even prefer online courses over using textbooks [25]. Having an e-learning package available will allow pre-clinical students to hone their interpretation skills in a risk free environment at their own pace in preparation for clinical work [13].

With the progress of modern technology, there has been a significant increase in the use of simulation technology for teaching and assessment in medical education, which has provided additional opportunities for learning around the compact curriculum [13]. E-learning modules have a number of advantages over the standard didactic approach that is familiar to most classrooms, such as the ability to learn at anytime and anywhere, without having to travel or take time off other commitments during business hours for live classes [15]. Simulation via e-learning also allows students to hone their skills in a risk-free environment [13], and the learner is able to spend more time on certain subjects or skip others that they already know [15], allowing an increased autonomy in the educational experience for the student. However, it is important that the actual content of the e-learning is interactive and attempts to simulate real clinical scenarios. In a recent study by Tan and colleagues [26], the authors implemented an e-learning program that was an online recording of lectures and compared the performance of this group with that of one who experienced didactic teaching in the classroom. In this study, students scored similar results in e-learning versus lectures. This suggests that just because content is made available online, it doesn’t necessarily make it a better teaching tool than if it was available directly. It is important that the actual content of the e-learning is addressed, ensuring that students are challenged to think clinically by the program and apply their knowledge.

## Conclusion

As more rural clinical schools are established in Australia and New Zealand, and the participation in courses by correspondence becomes anecdotally more popular with students, the electronic delivery of medical and health teaching will become a great priority [27]. The results of this study demonstrate that implementing radiological education through electronic means improved knowledge and skills in X-ray interpretation for students who participated in the online learning tool. Year 1 students showed a statistically significant improvement in X-ray interpretation skills following implementation of an e-learning x-ray interpretation package. Assessment of the post-intervention Year 1 students also showed scores that were statistically significantly better than the scores of Year 2 students who did not receive the intervention. With the exception of access to the online e-learning tool, the Year 2 students teaching and learning experience was the same as that of the Year 1 students. Further research is required to determine if the improved skills in X-ray interpretation is sustained in those students who have benefitted from the e-learning tool.
